# Complete Genome Sequence of Lactobacillus curvatus NFH-Km12, Isolated from the Japanese Traditional Fish Fermented Food Kabura-zushi

**DOI:** 10.1128/MRA.00823-18

**Published:** 2018-07-12

**Authors:** Daisuke Kyoui, Natsumi Mikami, Hiroyuki Yamamoto, Taketo Kawarai, Hirokazu Ogihara

**Affiliations:** a Laboratory of Food Hygiene, Department of Food Bioscience and Biotechnology, College of Bioresource Sciences, Nihon University, Fujisawa, Kanagawa, Japan; Louisiana State University

## Abstract

Kabura-zushi is a traditional Japanese fermented food made from yellowtail, rice, salt, and kōji. In this study, the complete genomic sequence of Lactobacillus curvatus NFH-Km12, isolated from this unique food, is reported. NFH-Km12 has a 1.9-Mbp chromosome and contains 5 plasmids.

## Abstract (precis)

Kabura-zushi is a traditional Japanese fermented food made from yellowtail, rice, salt, and kōji. In this study, the complete genomic sequence of Lactobacillus curvatus NFH-Km12, isolated from this unique food, is reported.

## ANNOUNCEMENT

Lactobacillus curvatus is a lactic acid bacterium widely associated with food fermentation (1–3). L. curvatus has also been isolated from kabura-zushi, a traditional Japanese fermented food made from salted yellowtail, salted turnip, and kōji (malted rice) (4). Because of the unique environment produced through the combined fermentation of fish and vegetables, it was expected that L. curvatus from kabura-zushi would have a unique phenotype or genotype. To explore its genetic characteristics, L. curvatus NFH-Km12, isolated from kabura-zushi, was sequenced.

NFH-Km12 was cultured by Lactobacillus de Man-Rogosa-Sharpe (MRS) broth (Becton, Dickinson and Company, NJ, USA) under 30°C overnight and provided for DNA extraction by the phenol-chloroform method. Whole-genome sequencing of NFH-Km12 was carried out using Illumina MiSeq and Oxford Nanopore minION platforms. For sequencing, from almost 1 µg of extracted DNA, 2 × 300-bp paired-end libraries and around 10-kbp sheared libraries prepared using g-TUBE (Covaris, Inc., MA, USA) were used for MiSeq and minION sequencing, respectively. Hybrid *de novo* assembly of the 1.41 million reads from MiSeq sequencing and 82,421 reads from minION sequencing that were expected to have ∼540× coverage was performed using Unicycler (5). Six circular contigs that were indicated as complete sequences by Unicycler were obtained from the assembly and were annotated using DFAST (6). The strain contained a 1,911,461-bp chromosome with a G+C content of 42.00% and 6 rRNA operons, 65 tRNAs, and 1,946 coding sequences (CDSs). The 5 remaining contigs were determined to be plasmids (pNFH-Km12A, 37,398 bp; pNFH-KM12B, 21,680 bp; pNFH-Km12C, 12,250 bp; pNFH-Km12D, 3,029 bp; pNFH-Km12E, 2,023 bp), with a total of 86 CDSs, making the isolate unlike previously reported L. curvatus strains.

### Data availability.

This whole-genome shotgun project has been deposited in ENA under BioProject number PRJDB6985, and the assembly has been deposited in GenBank under the accession numbers AP018699 to AP018704.
